# A Comparison of 3D Conformal and Deep Inspiratory Breath Holding vs. 4D-CT Intensity-Modulated Radiation Therapy for Patients with Left Breast Cancer

**DOI:** 10.3390/cancers15245799

**Published:** 2023-12-11

**Authors:** Moustafa Aldaly, Azza Hussien, Inas Mohsen El-nadi, Nabila Ibrahim Laz, Amira S. A. Said, Mohammad M. Al-Ahmad, Raghda R. S. Hussein, Al Shaimaa Ibrahim Rabie, Ahmed Hassan Shaaban

**Affiliations:** 1 Department of Clinical Oncology, Faculty of Medicine, Kasr AL Ainy, Cairo University, Cairo 11956, Egypt; moustafa.aldaly@kasralainy.edu.eg; 2 Department of Clinical Oncology, Faculty of Medicine, Beni-Suef University, Beni Suef 62511, Egypt; azza.husseinbakry@med.bsu.edu.eg (A.H.); drenas.mohsen@med.bsu.edu.eg (I.M.E.-n.); ahmed.hassanshaban@med.bsu.edu.eg (A.H.S.); 3 Department of Chest, Faculty of Medicine, Beni-Suef University, Beni Suef 62511, Egypt; nabila.ibrahim@med.bsu.edu.eg; 4 Department of Clinical Pharmacy, Faculty of Pharmacy, Beni-Suef University, Beni Suef 62511, Egypt; amira.ahmed@aau.ac.ae; 5 Department of Clinical Pharmacy, College of Pharmacy, Al Ain University, Abu Dhabi P.O. Box 112612, United Arab Emirates; 6 Department of Clinical Pharmacy, Faiyum Oncology Center, Faiyum 63511, Egypt; 7 Department of Clinical Nutrition, Health Insurance Authority, Faiyum 63511, Egypt

**Keywords:** breast irradiation, adjuvant radiotherapy, DIBH, RPM, 4D-CT IMRT, cardiac toxicity

## Abstract

**Simple Summary:**

Using four-dimensional computer tomography (4D-CT) or three-dimensional CT (3D-CT) simulation in breast cancer radiation is still debatable. This is a prospective feasibility trial for left breast cancer patients. All received adjuvant locoregional radiotherapy after either breast conservative surgery (BCS) or modified radical mastectomy (MRM) was used to compare the 4D-CT IMRT radiotherapy plan to the 3D conformal with the DIBH plan. A comparison was made regarding the dosimetry of the target and organ at risk (OAR). The 4D-CT IMRT plan is an acceptable alternative to the 3D conformal with the DIBH technique in patients meeting the exclusion criteria of the DIBH maneuver. The dosimetry of MHD (mean heart dose) in the 4D-CT IMRT plan was significantly lower than in the 3D conformal with the DIBH plan, and there was no significant difference between the two plans regarding mean LAD (left anterior descending artery). Further, a follow-up is recommended to assess late toxicity, especially regarding organs at risk (OAR) and the relapse rate.

**Abstract:**

Background: Multimodality is required for the treatment of breast cancer. Surgery, radiation (RT), and systemic therapy were traditionally used. Pharmacotherapy includes different drug mechanisms, such as chemotherapy, hormone therapy, and targeted therapies, alone or in combination with radiotherapy. While radiation offers numerous benefits, it also has certain harmful risks. such as cardiac and pulmonary toxicity, lymphedema, and secondary cancer. Modern radiation techniques have been developed to reduce organs at risk (OAR) doses. Materials and Methods: This study is a prospective feasibility trial conducted at the Fayium Oncology Center on patients with left breast cancer receiving adjuvant locoregional radiotherapy after either breast conservative surgery (BCS) or modified radical mastectomy (MRM). This study aimed to assess the proportion of patients who are fit both physically and intellectually to undergo breast radiotherapy using the deep inspiratory breath-holding (DIBH) technique, comparing different dosimetric outcomes between the 3D dimensional conformal with DIBH and 4D-CT IMRT plans of the same patient. Results: D95 of the clinical target volume (CTV) of the target is significantly higher in the 3D DIBH plan than in the IMRT plan, with an average of 90.812% vs. 86.944%. The dosimetry of the mean heart dose (MHD) in the 4D-CT IMRT plan was significantly lower than in the 3D conformal with the DIBH plan (2.6224 vs. 4.056 Gy, *p* < 0.0064), and no significant difference between the two plans regarding mean left anterior descending artery (LAD) (14.696 vs. 13.492 Gy, *p* < 0.58), maximum LAD (39.9 vs. 43.5 Gy, *p* < 0.35), and V20 of the ipsilateral lung (18.66% vs. 16.306%, *p* < 0.88) was observed. Internal mammary chain (IMC) irradiation was better in the 4D-CT IMRT plan. Conclusions: Radiotherapy of the breast and chest wall with the 4D-CT IMRT technique appears not to be inferior to the 3D conformal with the DIBH technique and can be used as an alternative to the 3D conformal with the DIBH technique in patients meeting the exclusion criteria for performing the DIBH maneuver concerning coverage to target volumes or unacceptably high doses to OAR.

## 1. Introduction

Breast cancer is the most prevalent malignancy, with an estimated two million new cases being identified each year worldwide [1]. In the United States, breast cancer is the most common cancer and the second leading cause of cancer death in women [2]. Fewer individuals have died from breast cancer since the 1970s [3]. This decrease in mortality is due to increased breast cancer screening and improvements in adjuvant therapy [4,5]. The treatment of breast cancer has various multimodal protocols. Surgery, radiation therapy (RT), and systemic therapy were traditionally used. Pharmacotherapy includes different drug mechanisms, such as chemotherapy, hormone therapy, and targeted therapies, alone or in combination with radiotherapy [6]. Breast conservation is now possible in some situations owing to radiotherapy’s growing role in the treatment of breast cancer. The radiotherapy improved the control of locoregional variables and survival for women who undergo mastectomy and are at high risk of recurrence [7]. Radiation therapy has beneficial effects, but also exhibits some healthy risks, such as harmful cardiac and lung effects, fluid retention, and secondary malignancy. It has been acknowledged that cardiac injury during breast cancer radiation is a significant problem. Patients who had adjuvant radiation had an increased chance of dying from heart disease (rate ratio of 1.27), according to a meta-analysis by the Early Breast Cancer Trialists’ Collaborative Group (EBCTCG) that included 78 randomized studies [8]. During radiotherapy for breast cancer, the heart is exposed to ionizing radiation, which raises the risk of subsequently developing ischemic heart disease. Within a few years of exposure, a spike starts, and it lasts for at least 20 years. It is proportional to the mean dosage of the heart. Radiotherapy poses a more considerable absolute risk to women with pre-existing cardiac risk factors than to other women [9]. Dosimetric consensus constraints for the heart system and its substructure are still being assessed and developed. The probability of radiation-induced cardiac death at ten years appears very low if the mean heart dose (MHD) is less than 3.3 Gy and the maximum left anterior descending artery (LAD) dosage (EQD23 Gy) is less than 45.4 Gy. This is stated in the retrospective case–control study by Beaton [10]. Radiation pneumonia is directly linked to the amount of lung exposure to radiation during tangential fields, the use of an additional supraclavicular (SC) field, and prior chemotherapy exposure (Anthracyclines, Taxanes). Moreover, high-dose chemotherapy, concurrent tamoxifen use, and smoking habits have all been linked to radiation pneumonitis [11]. Radiotherapy (RT) technology has significantly improved over recent decades. The purpose of implementing 3D planning radiotherapy and contemporary methods such as intensity-modulated radiation therapy (IMRT) and volumetric-modulated arc therapy (VMAT) has been to improve target coverage and spare vital organs. The precision of RT delivery has significantly increased due to the inclusion of numerous imaging modalities, contemporary computer technology, and linear accelerator (Linac) technological advancements in treatment planning [12]. Herein, this is a feasibility trial for patients with breast cancer receiving adjuvant locoregional radiotherapy after either breast conservative surgery (BCS) or modified radical mastectomy (MRM) aimed at the assessment of the proportion of patients who undergo breast radiotherapy using the DIBH technique, and by performing a dosimetric outcome comparison between the 3D conformal with DIBH and 4D-CT IMRT plans of the same patient. 

## 2. Materials and Methods

### 2.1. Setting Study Design

This study is a prospective feasibility trial conducted at the Fayium Center of Clinical Oncology for patients with left breast cancer receiving adjuvant locoregional radiotherapy after either breast conservative surgery (BCS) or modified radical mastectomy (MRM). This research aims to measure the proportion of patients who were physically fit (no comorbidities such as chest diseases or hearing problems) and intellectually fit (can understand the maneuver and communicate with the doctor and the technician). All the eligible patients underwent breast radiotherapy using the DIBH technique. A dosimetric outcome comparison was conducted between the 3D conformal with DIBH and 4D-CT IMRT plans of the same patient. The treatment session using the DIBH technique was time-consuming. Three-dimensional conformal planning was performed utilizing the Eclipse version 15.6 using AAA algorithm (V.15.0.31), two tangential fields with field-in-field technique were used for the breast and chest wall fields, and weighted antro-posterior fields were used for the paraclavicular fields. Moreover, one isocenter for the chest wall, breast and paraclavicular lymph nodes with a half-beam block was utilized.

Intensity-modulated radiotherapy (IMRT) using 5 to 7 fields covering both the chest wall, breast, and target lymph nodes with a dose rate of 600 Mu/min as performed. Plans were completed with A × B algorithm (V.15.0.31) with a calculation grid size of 0.25 cm. The optimization was carried out using a progressive resolution optimizer (PRO) (V.15.0.2.31). The prescribed dose was either 45 Gy over 20 fractions for 4 weeks for the chest wall, breast, and lymph nodes or a sequential boost was added to the tumor bed in breast conservative cases with a rate of 10 Gy over 5 fractions for 1 week [13].

### 2.2. Subjects

Forty patients were eligible and enrolled in this study. Patients were taught to perform respiratory movements using the thoracic rather than the abdominal muscles. For DIBH treatment sessions, they were required to take three full breaths, each with a shallow breath in and a prolonged breath out, to allow CO_2_ to be expired. This allowed for a longer breath hold in the deep inspiratory breath that followed. After that, patients were given incentive spirometers to carry out breathing exercises. This was intended to allow for more significant lung expansion and increased breath hold duration. After a few days of breathing exercising, the patients were brought in to assess their ability to follow instructions for performing the breath hold [14]. The intellectual fitness assessment was used as curated upon receiving the start signal. If successful, the physical fitness assessment was evaluated for their ability to hold their breath for 15–20 s. Upon proving their competence in the previous steps, the patients’ ability to generate a reproducible DIBH was assessed on the real-time position management (RPM) system during a CT simulation [15]. Each patient was then sent for a CT simulation. A breast board was used for each patient to keep the target volume flat and avoid oblique incidence. Each patient was instructed to hold the column using both hands. In paraclavicular lymph node irradiation cases, patients are advised to tilt the head to the opposite side of irradiation while lowering the arm on the involved side as much as possible to allow for maximum exposure to the irradiated fields [16,17]. CT reference markers were placed at the level of the xiphisternal junction in the midline and mid-axillary planes. Additional radio-opaque features were placed on the patients’ mastectomy or conservative surgery scar. Tumor bed markers were added in case of breast conservation [16]. After calibration of the infrared camera of the Varian RPM system, the external fiducial marker (EFM) box, which acts as a surrogate for respiratory motion, was placed on each patient’s xiphoid [18]. The process allowed for maximum motion range with each breath. The camera detected the EFM and calculated the position and movement of the thorax accordingly [19]. Upon success in the previous steps, CT imaging was performed using the Canon Aquilion lightning 160 (respiratory correlated multi-sliced CT) starting at the level of C3-4 and ending at below costophrenic angles mid-abdominal, with 3 mm slice thickness. On the other hand, DIBH, another 16-slice CT scanner, acquired the free-breathing 4D-CT scans. The Varian real-time position management (RPM) system (Varian Medical Systems, Palo Alto, CA, USA) was used to record the respiratory signal by tracking the movement of infrared markers placed on each patient’s epigastric abdomen area [20]. The reconstructed 4D-CT images were sorted using G.E. Advantage 4D software (G.E. Healthcare, Waukesha, WI, USA) into ten respiratory phases with labels ranging from 0% to 90%. Maximum end inspiration (EI) was represented by phase 0%, and maximum end expiration was characterized by phase 50% (EE). This study was used for structure delineation and the production of treatment plans; the 4D-CT images were reconstructed using a 2 mm thickness and then imported into the Eclipse treatment planning system (TPS) (Eclipse 15.6, Varian Medical Systems, PaloAlto, Santa Clara, CA, USA) for target delineation (chest wall, whole breast, paraclavicular lymph nodes, IMC, axillary LN) and organs at risk (heart, I-L, LAD, esophagus, and CLB) according to RTOG guidelines and assessment of D90, D95 of the target and MHD, V20 of I-L Dmean and Dmax of LAD, and V3 of CLB and Dmean of the cervical esophagus [14].

### 2.3. Inclusion Criteria

The eligible patients were 18 to 70 years old. Patients older than 70 years might be unable to perform the maneuver, and we mostly omitted radiotherapy. The inclusion criteria included pathologically proven LT breast cancer with stage I–III (AJCC Stage T1–4, N1–3). Both unilateral or bilateral (synchronous or metasynchronous) patients with breast cancer were enrolled. Also, surgical resection of breast cancer, either conservative (namely lumpectomy or quadrantectomy) or modified radical mastectomy, was included. All study participants provided written informed consent, including patients with compensated cardiac disease indicated for breast irradiation.

### 2.4. Exclusion Criteria

The exclusion criteria included age below 18 or above 70 years. Metastatic disease, locally recurrent disease, and pregnancy were excluded. Patients with severe active comorbidity factors (e.g., congestive heart failure, chronic obstructive pulmonary disease, or hepatic insufficiency) were excluded. Also, our study did not include patients who could not follow instructions for DIBH training or hold their breath for 15 to 20 s after training for a week. They were excluded if the patient’s breath curve amplitude on RPM was irregular and irreproducible. Moreover, the patient whose DIBH curve amplitude on RPM was less than 3 mm above the FB curve was excluded. Finally, patients with hearing problems were not enrolled.

### 2.5. Primary Outcomes

Dosimetric Assessment: Two plans were performed for each patient, one for the DIBH images set and another project with the 4D IMRT maximal intensity projection (MIP plan). Then, dosimetric differences between both scenarios were assessed regarding the target coverage and OAR protection. Session duration was recorded for each patient, starting from the end of the patient setup on the treatment table until the end of beam-on time. This study assessed the time needed to apply the DIBH technique for each patient. The median session duration was calculated for each week and was compared to the values of successive weeks for each patient.

### 2.6. Data Collection

Data were entered into the computer, and statistical procedures were used to analyze the data using STATA 14.2 (College Station, TX, USA). Numbers and percentages were used to describe qualitative data. The normality of the distribution was examined using the Kolmogorov–Smirnov test. The range (minimum and maximum), mean, standard deviation, median, and interquartile range (IQR) were used to explain the quantitative data. The 5% significance level was used to determine the results’ significance.

### 2.7. Statistical Analysis of the Data

All data were tabulated and statistically studied with descriptive analysis. A comparative dosimetric and geometric data analysis was performed using paired Student’s *t*-tests. Univariate and multivariate analysis was performed using Pearson’s coefficient and linear regression. A probability value (*p*-value) less than 0.05 was considered significant; all *p*-values were two-sided. The statistical calculations were performed using STATA 14.2 (College Station, TX, USA).

## 3. Results

### 3.1. Baseline Characteristics

Forty patients were screened for the feasibility of DIBH planning; twenty-five patients performed the technique and were included in the comparative analysis. The median age was 45 years, ranging from 24 to 68 years; all patients were non-smokers. Of the 15 excluded patients, 5 of them were old and intolerant (above 60 years), 5 had chronic chest diseases (mostly asthmatic patients), 2 had hearing problems, and 3 patients had poor communication with the doctor and technician. They could not understand the maneuver and were phobic of the machine. Table 1 summarizes the baseline characteristics of our cohort.

### 3.2. Dosimetric Parameters

**Figure 1:**
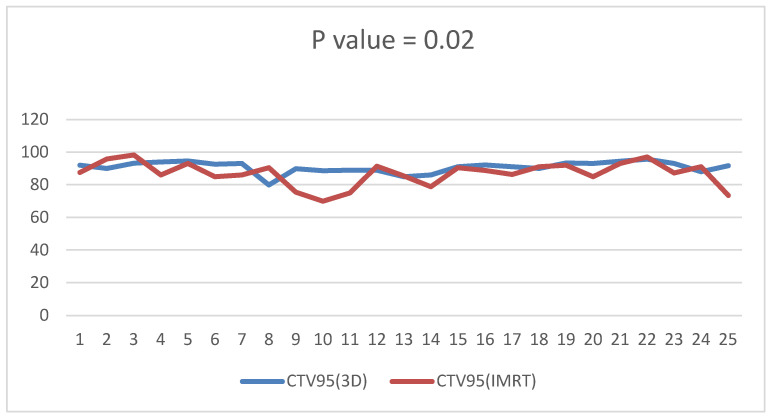
D95 of the CTV of the target is significantly higher in the DIBH plan than the IMRT plan with a *p* value < 0.02.

**Figure 2:**
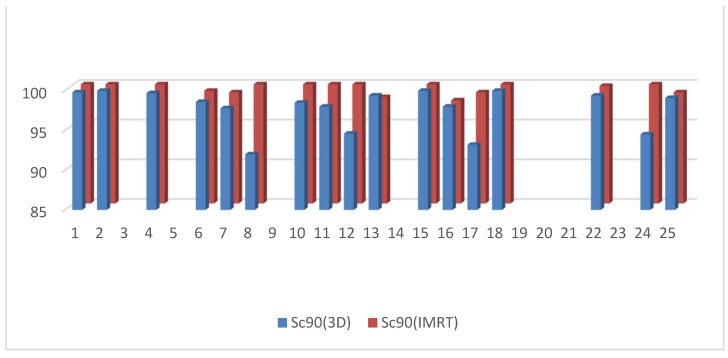
D90 of the CTV of the supraclavicular LNS in the 3D DIBH plan and the IMRT plan.

**Figure 3:**
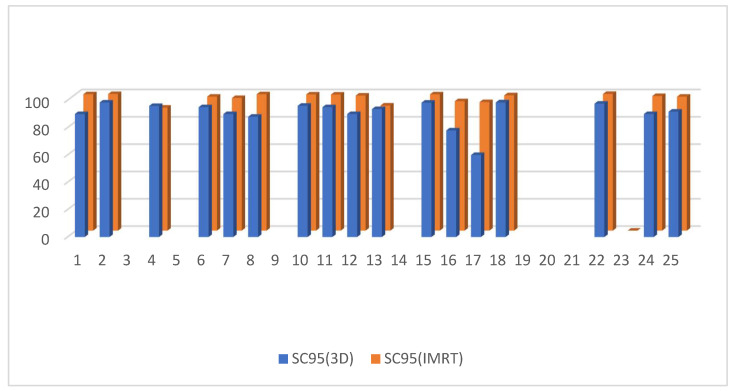
D95 of the CTV of the of the Supraclavicular LNS in the 3D DIBH and IMRT plans.

**Figure 4:**
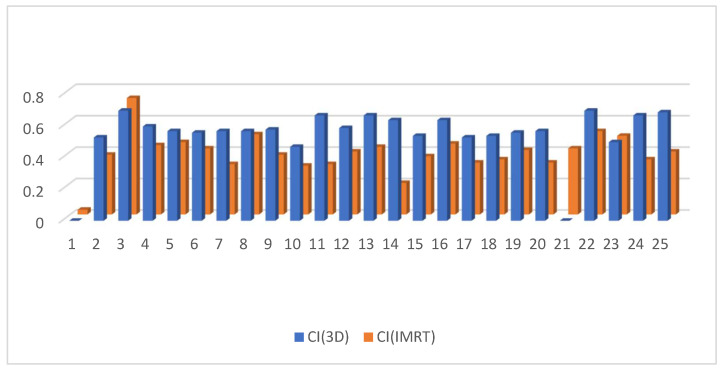
CI in the 3D conformal with the DIBH versus IMRT plans.

**Figure 5:**
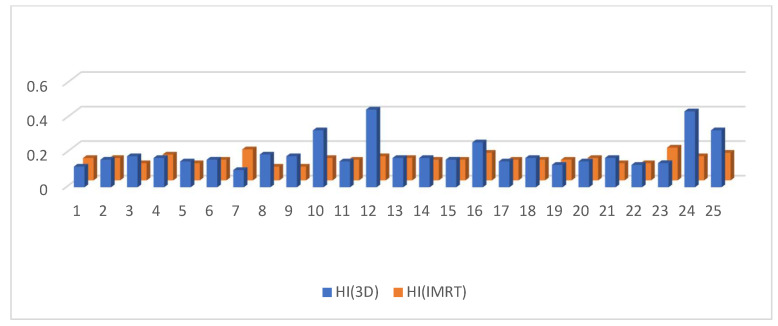
HI in the 3D conformal with the DIBH plan versus the IMRT plan.

D95 of the clinical target volume of the target was significantly higher in the 3D DIBH plan than in the IMRT plan, with an average of (90.812 ± 3.48 vs. 86.944 ± 7.37) shown in Figure 1. Only 17 patients underwent supraclavicular LNS radiation; there was a significant difference between the two plans in D90 and D95 of the CTV of the supraclavicular LNS, with more coverage in the 4D IMRT plan than the DIBH plan (Figure 2 and Figure 3). Moreover, the conformity index (CI) and homogeneity index (HI) were significantly higher in the 3D conformal with the DIBH plan than in the IMRT plan, as shown in Figure 4 and Figure 5. 

Interestingly, the D95 of the CTV of the paraclavicular LNS was significantly higher in the IMRT plan than the 3D DIBH plan (average 97.5176 ± 3.1 vs. 90.7353 ± 9.3) *p*-value < 0.002, as shown in Figure 3.

Moreover, the conformity index (CI) and homogeneity index (HI) were significantly higher in the 3D conformal with the DIBH plan than in the IMRT plan, as in Figure 4 and Figure 5.

HI was considerably higher in the 3D conformal with the DIBH plan than the IMRT plan, as shown in Figure 5.

**Figure 6:**
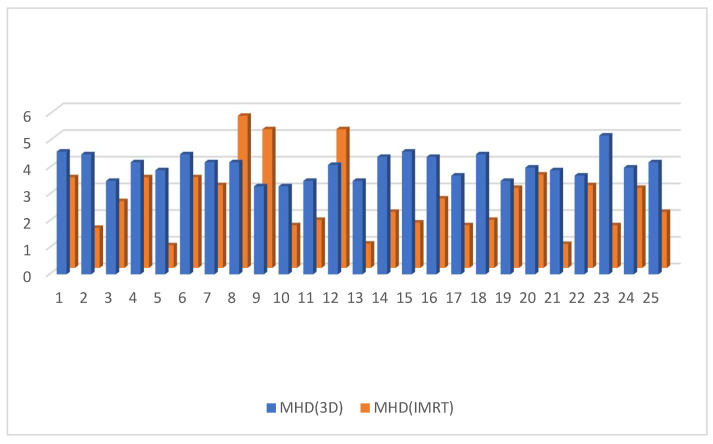
Mean heart dose in the IMRT plan versus the 3D conformal with the DIBH plan.

**Figure 7:**
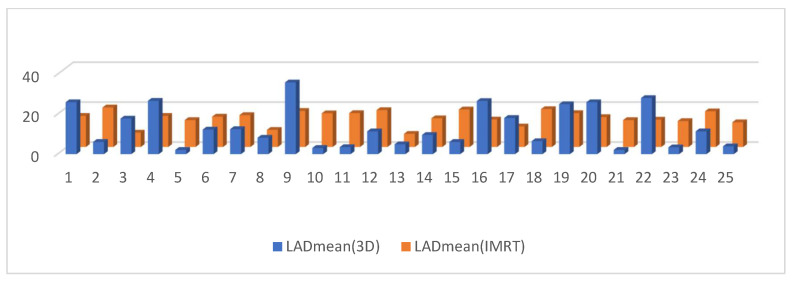
Mean LAD dose in the 3D conformal with the DIBH plan versus the IMRT plan.

**Figure 8:**
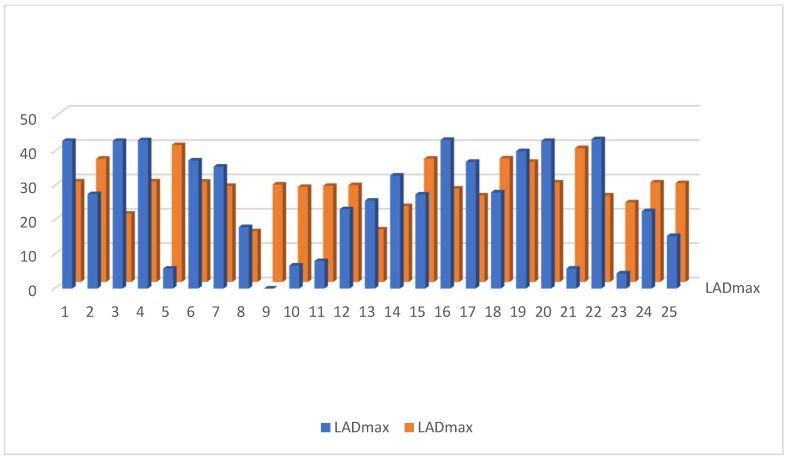
Maximum LAD dose in the 3D DIBH and IMRT plans.

The dosimetry of the mean heart dose (MHD) in the IMRT plan was significantly lower than in 3D DIBH plan—MHD (2.7224 ± 1.3 vs. 4.056 ± 0.47 G.Y.), *p*-value < 0.0064 (Figure 6.) Moreover, there was no significant difference in mean LAD dose between the IMRT plan and the 3D DIBH plan (14.696 ± 1.09 vs. 13.492 ± 3.5 Gy, *p* < 0.58), as shown Figure 7. The maximum LAD dose was insignificant in the 3D DIBH and IMRT (27.48 vs. 28.38) plans with a *p*-value of 0.35 (Figure 8). 

**Figure 9:**
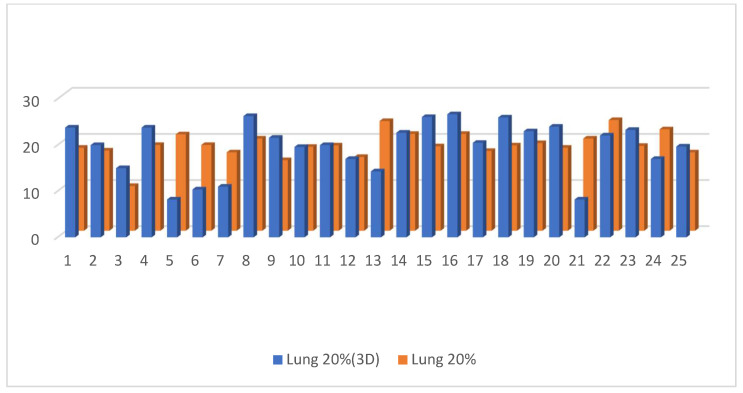
Dose of V20 of the ipsilateral lung in the 4D-CT IMRT plan and the 3D DIBH plan.

**Figure 10:**
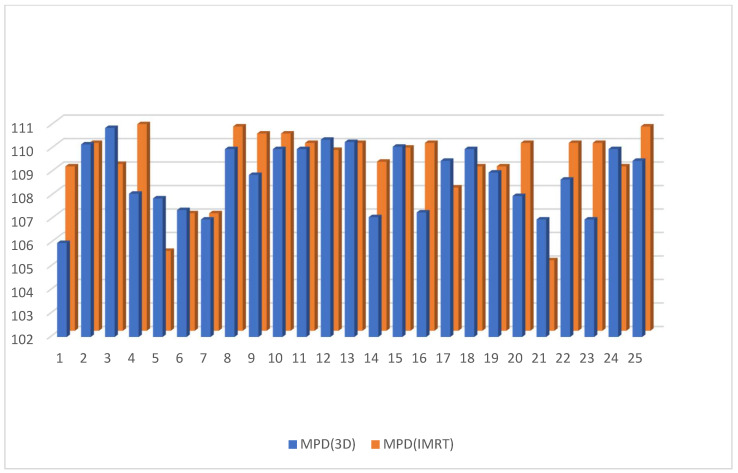
Maximum point dose in the 3D conformal with the DIBH plan versus the IMRT plan.

**Figure 11:**
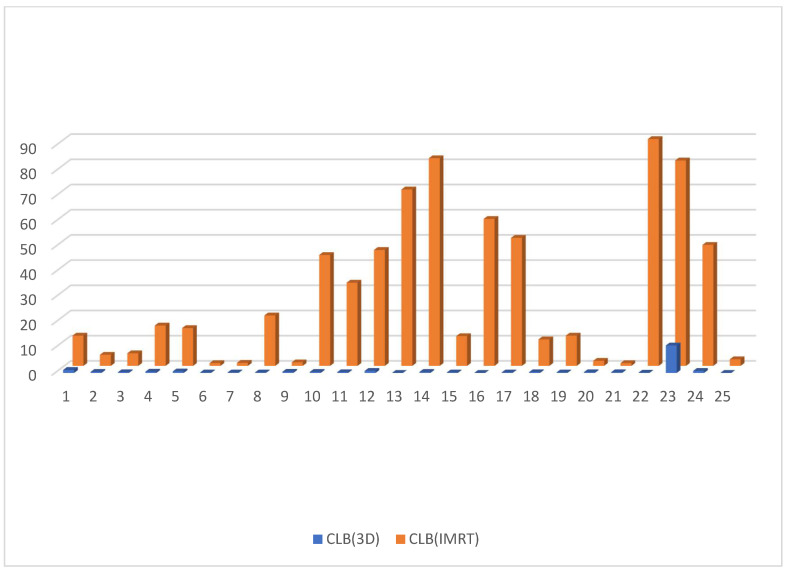
Comparing CLB in the DIBH plan versus the IMRT plan.

**Figure 12:**
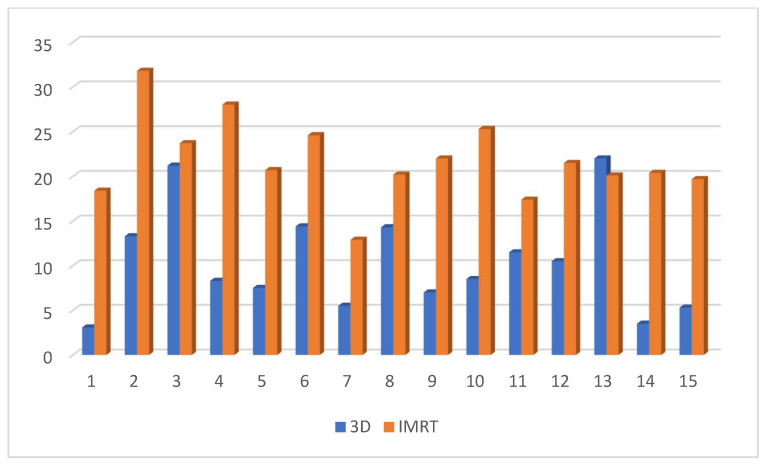
Mean dose comparison regarding the cervical esophagus between the two plans; *p* value < 0.00841.

Dose to V20 of the ipsilateral lung showed no significant difference between the 3D DIBH and IMRT (16% vs. 18%) plans (Figure 9). Also, the maximum point dose (MPD) was insignificant between the IMRT plan and the 3D DIBH plan (108.8 ± 1.4 vs. 109.1 ± 1.5), as shown in Figure 10. On the other hand, the mean amount to the contralateral breast (Figure 11) and cervical esophagus was significantly lower in the 3D DIBH plan than in the 4D-CT IMRT plan, which was performed in 15 patients, as shown in Figure 12. 

Finally, IMC irradiation was indicated only in five patients, and D95 was not significantly different between both plans but higher in the IMRT plan (68.4 ± 12.4 vs. 93.1 ± 7.9).

## 4. Discussion

The architecture of breast tissue exhibits geometric changes that may impact the distribution of doses. Radiation therapy for breast cancer is very challenging. Therefore, selecting an appropriate radiotherapy technique is critical for ensuring safe treatment delivery [21]. The dosimetric advantage of IMRT over 3D-CT has been shown in several investigations; some of these trials have revealed reduced doses to the ipsilateral lung, heart, and left anterior descending artery [22]. In this prospective study, we compared the 3D conformal with the DIBH approach, which is considered to be the standard technique for left breast irritation, with the 4D-CT IMRT maximal intensity projection (MIP) plan on patients with left-sided breast cancer [23]. Twenty-five patients were enrolled and performed the procedure while holding their breath for 15 to 20 s and demonstrating high compliance. Moreover, another simulation using 4D-CT and an IMRT plan was performed on the same patient and compared to the 3D conformal DIBH plan [21]. The meta-analysis by [24] was composed of 41 trials with a total of 3599 patients. There was a statistically significant difference and a considerable increase in lung volume. There is no clear advantage of DIBH versus free breathing (FB) for contralateral breast mean dosage. Thus, DIBH is the standard technique used in left-sided breast irradiation, but many patients display characteristics which exclude them from being included and prevents them from performing this technique. Here, in our study, a dosimetric comparison between the 3D conformal with DIBH vs. IMRT (MIP plan) was used as an alternative therapy. Recent research has shown that the movement of tumors and organs at risk (OAR) can easily impair the precision and efficiency of radiation in the chest and abdomen [19,25]. Four-dimensional (4D)-CT radiation is a contemporary technological advancement to address these issues. The 4D-CT method can produce more precise images of tumors and healthy organs than traditional scanning [19]. In Yanli Yan’s study [26], seven patients with remaining breast tissue underwent whole breast radiation using 3D-CT and 4D-CT simulations. Four different types of CT pictures were created for the 4D-CT plan, including images taken using the maximal intensity projection (MIP), average intensity projection (AIP), and photos taken at the end of inspiration and expiration of the clinical target volume (CTV) and planning target volume (PTV). The 3D-CT plan was slightly higher than the 4D-CT plan, which matched our findings. The volumes of the 4D-CT plan for the contralateral breast (C-B) were significantly lower than those of the 3D-CT plan. Also, the volumes of the 3D-CT and AIP plans were greater than those of the MIP plan for the ipsilateral lungs (I-L). The volumes of the MIP and AIP plans for the heart that received at least 40% of the prescribed dose (V40) and at least 30% of the prescribed dose (V30) were marginally lower than those of the 3D plan. In conclusion, compared to 3D-CT radiotherapy for breast cancer, 4D-CT radiotherapy based on the MIP and AIP plans offers a slightly smaller radiation area and a slightly higher CTV and PTV radiotherapy dosage. Consequently, the MIP and AIP plans reduce exposure to C-B radiation and increase cardiac and I-L sparing, but both plans were created using [11,27]. Reardon’s study [28] conducted a comparison of a 3D conformal deep inspiratory breath hold versus free-breathing intensity-modulated radiation therapy for the treatment of left-sided breast cancer; ten women with left-sided breast cancer underwent two computed tomography scans, a voluntary DIBH, and free breathing. The mean volumes of the left anterior descending coronary artery (LAD) of the heart, right breast, and entire lung were computed for patients who received doses ranging from 5% to 95% of the recommended dosage. The mean volumes of the heart and the LAD were lower (*p* < 0.05) in 3D-DIBH compared to our findings. 3D-DIBH reduced the mean dosage to the heart and LAD (*p* < 0.01). With dosage levels of 20% to 75%, the mean volumes of the entire lung were smaller in FB-IMRT (*p* < 0.05), but the mean dose was not different. The mean volumes of the right breast did not differ between doses. However, 3D-DIBH had a lower mean dose (*p* = 0.04). As an alternative to FB-IMRT, 3D-DIBH offers patients with left-sided breast cancer a clinically equal course of treatment while protecting vulnerable organs and being more easily implemented. In our study, the dosimetry of some organs at risk in the 4D IMRT plans was significantly lower than the 3D conformal with DIBH plans, including the mean heart dose, which is in concordance with Yanli Yan’s study [26]. Regarding 4D IMRT, our findings are contrast those found in Reardon and Hayden’s study, in which the mean LAD dose showed no significant difference between both the 3D conformal with the DIBH plan and the 4D IMRT plan in relation to the DIBH arm in comparison with the FB arm, and the maximum LAD dose showed no significant difference between the 3D DIBH and 4D-CT IMRT plans [22,28]. With regard to V3 of the contralateral breast, it was significantly lower in the 3D conformal with the DIBH plan than in the 4D IMRT plan, which contrasts Yanli Yan et al.’s study [26] and supports the findings of Reardon and Hayden’s study [28]. Moreover, the cervical esophagus was significantly higher in the 4D IMRT plan than in the 3D conformal plan, especially in patients with supraclavicular irradiation. In addition, more in-depth data were found in the previous literature search that matched our findings [13,29].

In Conway’s study [11], comparing locoregional radiation therapy for DIBH to FB, CT scans for 30 patients were planned for both DIBH and FB in 21 patients (70%) with DIBH compared to FB, and there was a 5% drop in the ipsilateral lung V20. The mean ipsilateral lung V20 decline ranged from 0 to 20% with DIBH, and the mean lung dose (MLD) was reduced by 3.4 Gy on average (−0.2 to 9.1; *p* < 0.001) [14], which contrasts our findings. In concordance to Reardon and Hayden’s study [22,26,28], our study has shown no significant difference in the mean dose to V20 of the ipsilateral lung in the 3D conformal with the DIBH plan compared to the 4D-CT IMRT plan. However, this conflicted Yanli’s study, proving that more sparing occurred with the MIP plan for the ipsilateral lungs (I-L) in his analysis [26].

In our study, the dose of CTV95 of the target and paraclavicular LNS in the 3D conformal with the DIBH plan was higher than the 4D IMRT plan with a *p*-value < 0.002, which is contrary to Majumdar’s study [30]. Regarding the PTV coverage, the 3D-CRT and IMRT plans were the same in the PTV Max and PTV Min doses.

In a study by Majumdar et al. [30], about 35 patients with left-sided breast cancer with MRM were subject to three distinct plans for adjuvant radiation which were developed utilizing 3D-CRT, IMRT, and VMAT, in which 50 Gy in 25 portions was the recommended dose. To investigate the dosimetric relevance, the plans for PTV_95_, homogeneity index (HI), and conformity index (CI) were compared. Findings indicated that both the VMAT and IMRT plans improved PTV_95%_ coverage and CI compared to 3D-CT, which was statistically significant regarding pairwise analysis. On the other hand, the HI difference showed no significance. 

In our study, the CI and HI were significantly higher in the 3D conformal with the DIBH plan than in the 4D IMRT plan. In contrast to our analysis, Majumdar et al. [30] reported the absence of a significant difference in HI between the 3D-CT and IMRT plans. It was also reported that CI was better in the IMRT plan. 

The aim of Figlia et al.’s [31] study was to determine the effect of adding internal mammary chain (IMC) irradiation to regional nodal irradiation (RNI) for patients with node-positive left-sided breast cancer and to compare the excess relative and absolute risks of radiation-induced lung cancer/BC and ischemic heart disease for intensity-modulated radiotherapy (IMRT) versus 3D conformal radiotherapy (3D-CRT). Each of the ten patients who underwent evaluation received four different treatment plans (3D-CRT and IMRT −/+ IMC). When both techniques were used, the outcome was that IMC irradiation was added to RNI, significantly increasing the dose exposure to the heart, lung, and contralateral breast. This resulted in a higher ERR for secondary lung cancer (58 vs. 44%, *p* = 0.002), contralateral BC (49 vs. 31%, *p* = 0.002), and ischemic heart disease (41 vs. 27%, *p* = 0.002, IMRT plan). In comparison to 3D-CRT, IMRT significantly reduced the mean cardiac and lung doses, lowering the ERR for major coronary events (64% with 3D-CRT vs. 41% with IMRT, *p* = 0.002) and the ERR for secondary lung cancer (75 vs. 58%, *p* = 0.004) in IMC radiation while having no discernible effect on the risks for secondary contralateral BC in Comparison to 3D-CT; thus, the adoption of IMRT appears beneficial.

In our study, the internal mammary chain irritation (IMC) was significantly higher in the 4D-CT IMRT plan, with less toxicity to the heart and lung than in the 3D DIBH plan (with *p*-value < 0.002), which supports the finding of Figlia’s study [31]. Finally, this study had some limitations, such as a small sample size. Therefore, a larger clinical trial is needed to confirm our findings.

## 5. Conclusions

The radiotherapy for the breast and chest wall with the 4D-CT IMRT technique appears not to be inferior to the 3D conformal with the DIBH technique and can be used as an alternative to the 3D conformal with the DIBH technique in patients meeting the exclusion criteria. A significantly lower MHD, no significant difference in the mean LAD, the maximum LAD and V20 of the I-L dose, and more coverage to the supraclavicular field and IMC was found. Moreover, the findings indicate that the 4D-CT IMRT technique is less time-consuming and enables more appropriate doses to be administered for the target volume (planning target volume), concerning the range to the target, in comparison to the unacceptably high quantities that are administered with other techniques, which could be harmful for organs at risk. However, longer follow-up durations are always beneficial for the assessment of late toxicity, especially for organs at risk and the relapse rate. They will further enhance the confidence in the use of such techniques.

## Figures and Tables

**Table 1:** Baseline clinical characteristics of the eligible patients.

Parameter	Total, *n* = 25 (%)
Median age (range)	45 (24–68)
Mean weight	79.6 kg
Mean BMI	31.4
Premenopausal	10
Postmenopausal	15
Education level	
Illiterate or low literacy	13
High literacy	12
T.N.M. Stage	
T1	8 (32)
T2	12 (48)
T3	3 (12)
T4	2 (8)
N0 N1	9 (36.7)
N2–3	8 (26.7)
Chemotherapy	
Adjuvant	20 (80)
Neoadjuvant	5 (20)
Trastuzumab	6 (24)
Surgery	
Breast conservative	10 (40)
Modified radical mastectomy	15 (60)
Radiotherapy	
Breast and boost	10 (40)
Breast and chest wall	3 (12)
Postmastectomy chest wall	15 (60)

T: tumor stage, N: number of lymph nodes involved (only present in 17 patients), M: metastasis.

## Data Availability

Data are available upon reasonable request from the corresponding author.
